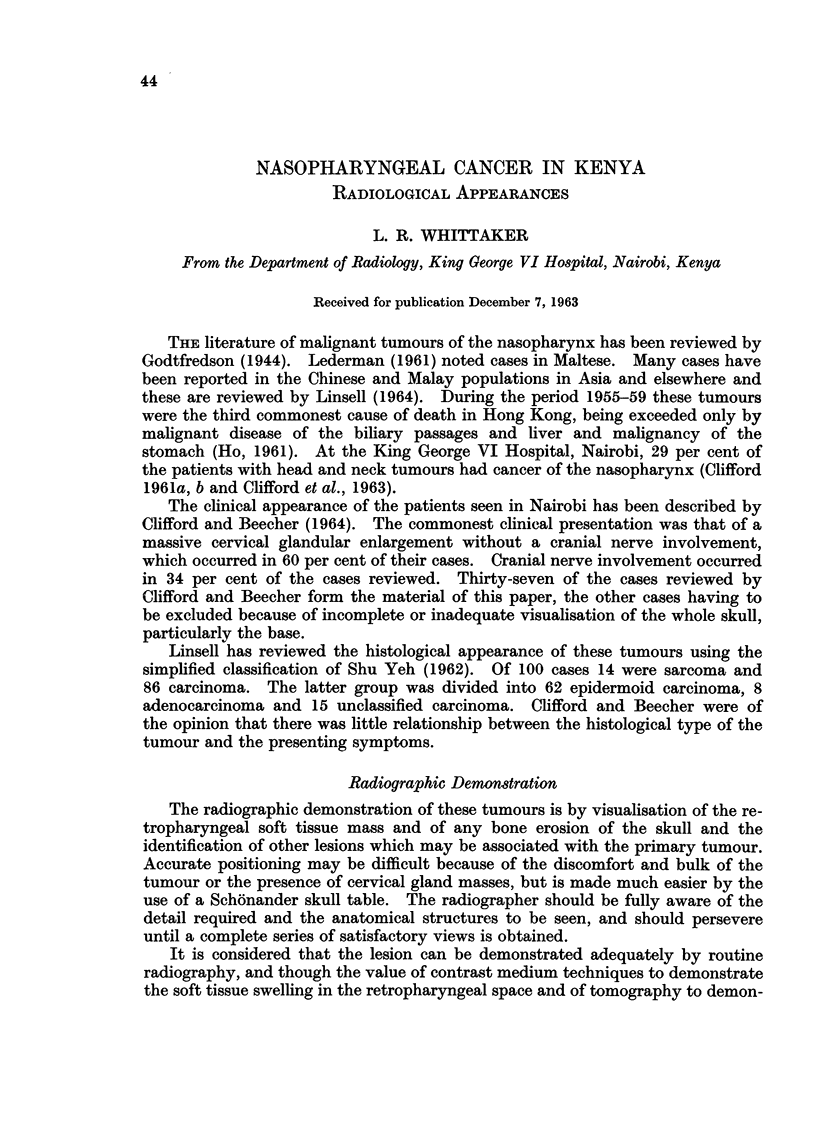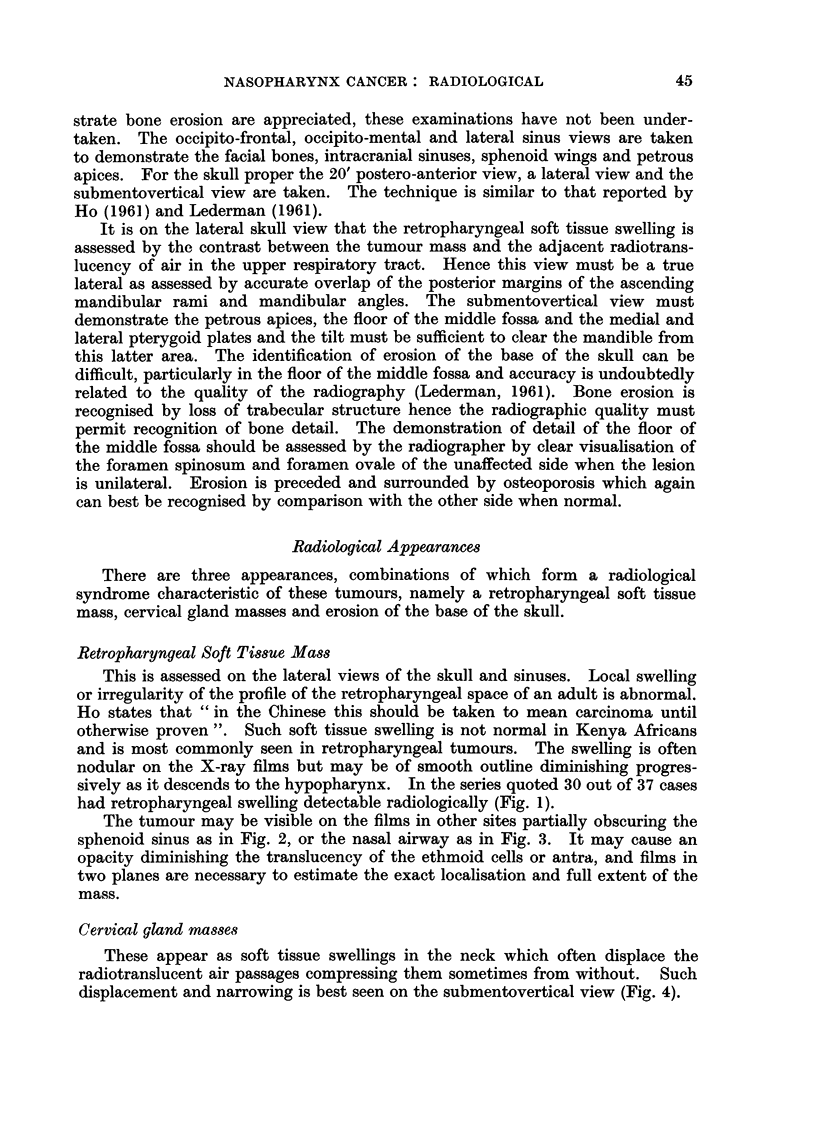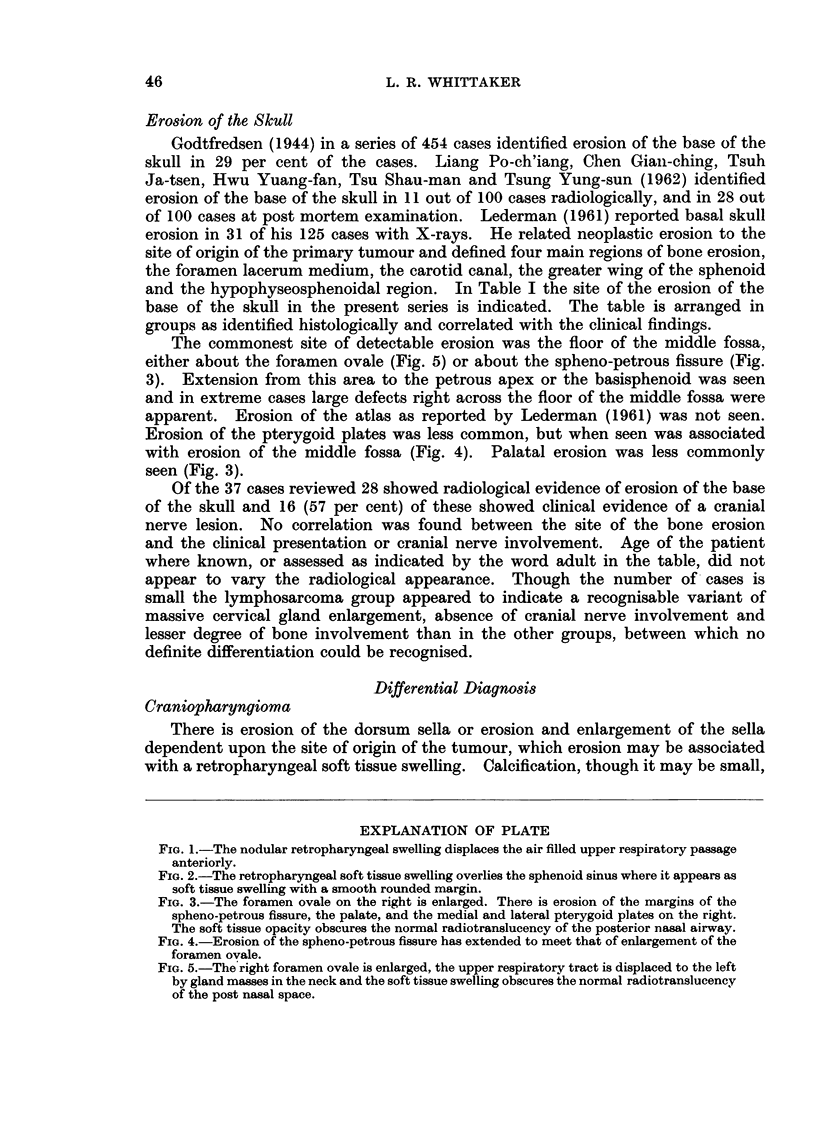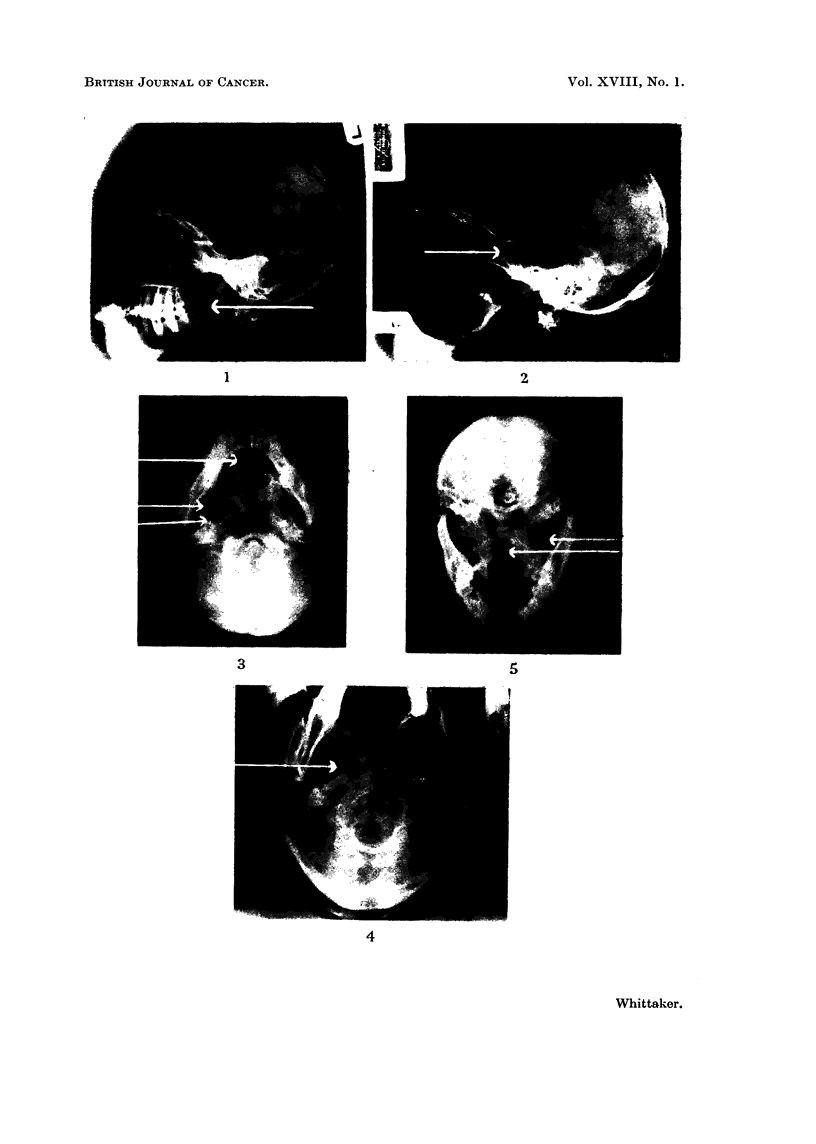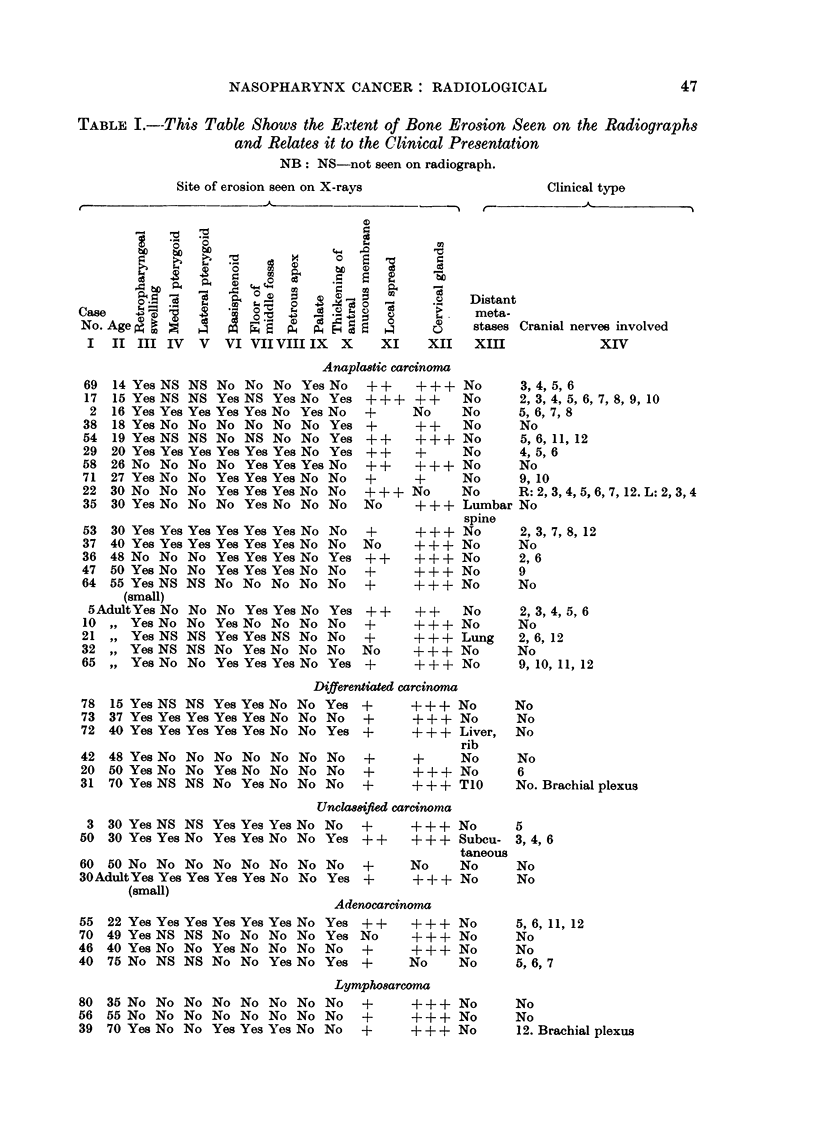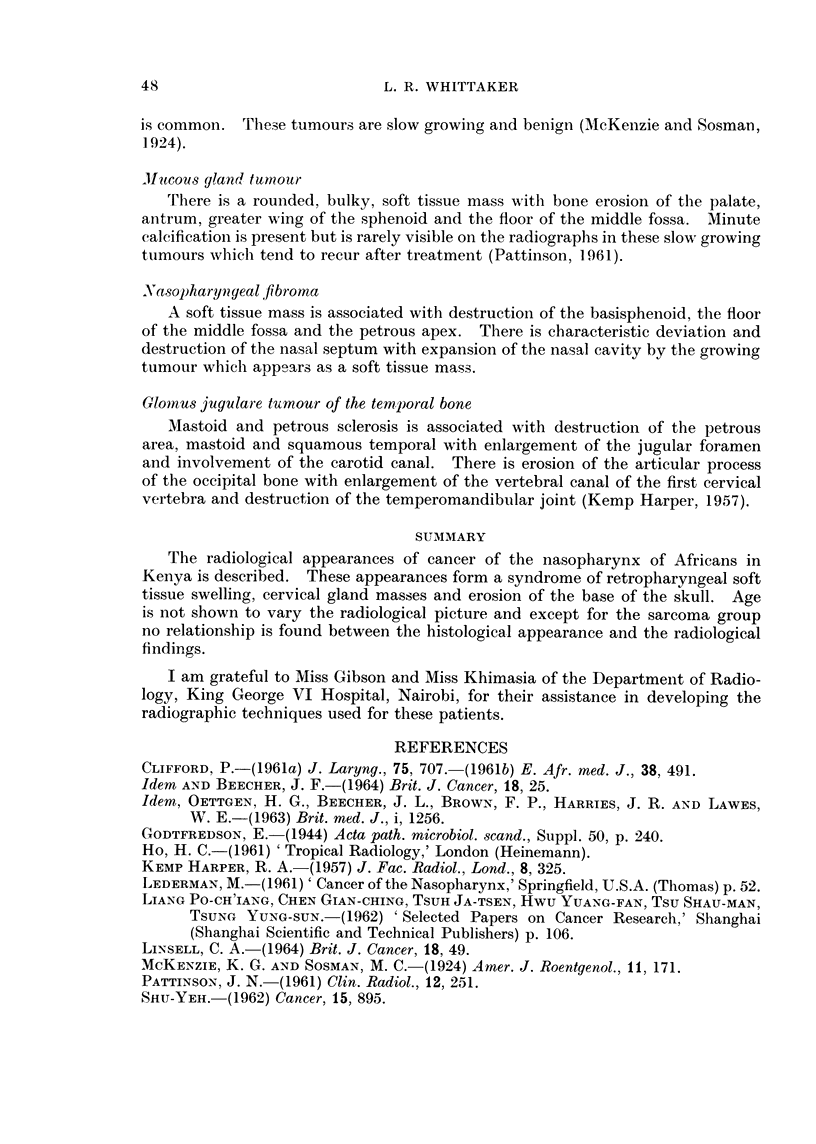# Nasopharyngeal Cancer in Kenya

**DOI:** 10.1038/bjc.1964.4

**Published:** 1964-03

**Authors:** L. R. Whittaker

## Abstract

**Images:**


### NASOPHARYNGEAL CANCER IN KENYA
### Radiological Appearances

L. R. WHITTAKER

*From the Department of Radiology, King George VI Hospital, Nairobi, Kenya*

Received for publication December 7, 1963

The literature of malignant tumours of the nasopharynx has been reviewed by Godtfredson (1944). Lederman (1961) noted cases in Maltese. Many cases have been reported in the Chinese and Malay populations in Asia and elsewhere and these are reviewed by Linsell (1964). During the period 1955–59 these tumours were the third commonest cause of death in Hong Kong, being exceeded only by malignant disease of the biliary passages and liver and malignancy of the stomach (Ho, 1961). At the King George VI Hospital, Nairobi, 29 per cent of the patients with head and neck tumours had cancer of the nasopharynx (Clifford 1961*a*, *b* and Clifford *et al.*, 1963).

The clinical appearance of the patients seen in Nairobi has been described by Clifford and Beecher (1964). The commonest clinical presentation was that of a massive cervical glandular enlargement without a cranial nerve involvement, which occurred in 60 per cent of their cases. Cranial nerve involvement occurred in 34 per cent of the cases reviewed. Thirty-seven of the cases reviewed by Clifford and Beecher form the material of this paper, the other cases having to be excluded because of incomplete or inadequate visualisation of the whole skull, particularly the base.

Linsell has reviewed the histological appearance of these tumours using the simplified classification of Shu Yeh (1962). Of 100 cases 14 were sarcoma and 86 carcinoma. The latter group was divided into 62 epidermoid carcinoma, 8 adenocarcinoma and 15 unclassified carcinoma. Clifford and Beecher were of the opinion that there was little relationship between the histological type of the tumour and the presenting symptoms.

### *Radiographic Demonstration*

The radiographic demonstration of these tumours is by visualisation of the retropharyngeal soft tissue mass and of any bone erosion of the skull and the identification of other lesions which may be associated with the primary tumour. Accurate positioning may be difficult because of the discomfort and bulk of the tumour or the presence of cervical gland masses, but is made much easier by the use of a Schönander skull table. The radiographer should be fully aware of the detail required and the anatomical structures to be seen, and should persevere until a complete series of satisfactory views is obtained.

It is considered that the lesion can be demonstrated adequately by routine radiography, and though the value of contrast medium techniques to demonstrate the soft tissue swelling in the retropharyngeal space and of tomography to demonstrate bone erosion are appreciated, these examinations have not been undertaken. The occipito-frontal, occipito-mental and lateral sinus views are taken to demonstrate the facial bones, intracranial sinuses, sphenoid wings and petrous apices. For the skull proper the 20′ postero-anterior view, a lateral view and the submentovertical view are taken. The technique is similar to that reported by Ho (1961) and Lederman (1961).

It is on the lateral skull view that the retropharyngeal soft tissue swelling is assessed by the contrast between the tumour mass and the adjacent radiotranslucency of air in the upper respiratory tract. Hence this view must be a true lateral as assessed by accurate overlap of the posterior margins of the ascending mandibular rami and mandibular angles. The submentovertical view must demonstrate the petrous apices, the floor of the middle fossa and the medial and lateral pterygoid plates and the tilt must be sufficient to clear the mandible from this latter area. The identification of erosion of the base of the skull can be difficult, particularly in the floor of the middle fossa and accuracy is undoubtedly related to the quality of the radiography (Lederman, 1961). Bone erosion is recognised by loss of trabecular structure hence the radiographic quality must permit recognition of bone detail. The demonstration of detail of the floor of the middle fossa should be assessed by the radiographer by clear visualisation of the foramen spinosum and foramen ovale of the unaffected side when the lesion is unilateral. Erosion is preceded and surrounded by osteoporosis which again can best be recognised by comparison with the other side when normal.

### *Radiological Appearances*

There are three appearances, combinations of which form a radiological syndrome characteristic of these tumours, namely a retropharyngeal soft tissue mass, cervical gland masses and erosion of the base of the skull.

### *Retropharyngeal Soft Tissue Mass*

This is assessed on the lateral views of the skull and sinuses. Local swelling or irregularity of the profile of the retropharyngeal space of an adult is abnormal. Ho states that " in the Chinese this should be taken to mean carcinoma until otherwise proven ". Such soft tissue swelling is not normal in Kenya Africans and is most commonly seen in retropharyngeal tumours. The swelling is often nodular on the X-ray films but may be of smooth outline diminishing progressively as it descends to the hypopharynx. In the series quoted 30 out of 37 cases had retropharyngeal swelling detectable radiologically (Fig. 1).

The tumour may be visible on the films in other sites partially obscuring the sphenoid sinus as in Fig. 2, or the nasal airway as in Fig. 3. It may cause an opacity diminishing the translucency of the ethmoid cells or antra, and films in two planes are necessary to estimate the exact localisation and full extent of the mass.

### *Cervical gland masses*

These appear as soft tissue swellings in the neck which often displace the radiotranslucent air passages compressing them sometimes from without. Such displacement and narrowing is best seen on the submentovertical view (Fig. 4).

*Erosion of the Skull*

Godtfredsen (1944) in a series of 454 cases identified erosion of the base of the skull in 29 per cent of the cases. Liang Po-ch'iang, Chen Gian-ching, Tsuh Ja-tsen, Hwu Yuang-fan, Tsu Shau-man and Tsung Yung-sun (1962) identified erosion of the base of the skull in 11 out of 100 cases radiologically, and in 28 out of 100 cases at post mortem examination. Lederman (1961) reported basal skull erosion in 31 of his 125 cases with X-rays. He related neoplastic erosion to the site of origin of the primary tumour and defined four main regions of bone erosion, the foramen lacerum medium, the carotid canal, the greater wing of the sphenoid and the hypophyseosphenoidal region. In Table I the site of the erosion of the base of the skull in the present series is indicated. The table is arranged in groups as identified histologically and correlated with the clinical findings.

The commonest site of detectable erosion was the floor of the middle fossa, either about the foramen ovale (Fig. 5) or about the spheno-petrous fissure (Fig. 3). Extension from this area to the petrous apex or the basisphenoid was seen and in extreme cases large defects right across the floor of the middle fossa were apparent. Erosion of the atlas as reported by Lederman (1961) was not seen. Erosion of the pterygoid plates was less common, but when seen was associated with erosion of the middle fossa (Fig. 4). Palatal erosion was less commonly seen (Fig. 3).

Of the 37 cases reviewed 28 showed radiological evidence of erosion of the base of the skull and 16 (57 per cent) of these showed clinical evidence of a cranial nerve lesion. No correlation was found between the site of the bone erosion and the clinical presentation or cranial nerve involvement. Age of the patient where known, or assessed as indicated by the word adult in the table, did not appear to vary the radiological appearance. Though the number of cases is small the lymphosarcoma group appeared to indicate a recognisable variant of massive cervical gland enlargement, absence of cranial nerve involvement and lesser degree of bone involvement than in the other groups, between which no definite differentiation could be recognised.

### *Differential Diagnosis*

*Craniopharyngioma*

There is erosion of the dorsum sella or erosion and enlargement of the sella dependent upon the site of origin of the tumour, which erosion may be associated with a retropharyngeal soft tissue swelling. Calcification, though it may be small,

---

EXPLANATION OF PLATE

Fig. 1.—The nodular retropharyngeal swelling displaces the air filled upper respiratory passage anteriorly.

Fig. 2.—The retropharyngeal soft tissue swelling overlies the sphenoid sinus where it appears as soft tissue swelling with a smooth rounded margin.

Fig. 3.—The foramen ovale on the right is enlarged. There is erosion of the margins of the spheno-petrous fissure, the palate, and the medial and lateral pterygoid plates on the right. The soft tissue opacity obscures the normal radiotranslucency of the posterior nasal airway.

Fig. 4.—Erosion of the spheno-petrous fissure has extended to meet that of enlargement of the foramen ovale.

Fig. 5.—The right foramen ovale is enlarged, the upper respiratory tract is displaced to the left by gland masses in the neck and the soft tissue swelling obscures the normal radiotranslucency of the post nasal space.

1

2

3

5

4

Whittaker.

TABLE I.—*This Table Shows the Extent of Bone Erosion Seen on the Radiographs and Relates it to the Clinical Presentation*

NB : NS—not seen on radiograph.

| | | Site of erosion seen on X-rays | | | | | | | | | | Clinical type | |
|---|---|---|---|---|---|---|---|---|---|---|---|---|---|
| Case No. | Age | Retropharyngeal swelling | Medial pterygoid | Lateral pterygoid | Basisphenoid | Floor of middle fossa | Petrous apex | Palate | Thickening of antral mucous membrane | Local spread | Cervical glands | Distant metastases | Cranial nerves involved |
| I | II | III | IV | V | VI | VII | VIII | IX | X | XI | XII | XIII | XIV |
| | | | | | | *Anaplastic carcinoma* | | | | | | | |
| 69 | 14 | Yes | NS | NS | No | No | No | Yes | No | ++ | +++ | No | 3, 4, 5, 6 |
| 17 | 15 | Yes | NS | NS | Yes | NS | Yes | No | Yes | +++ | ++ | No | 2, 3, 4, 5, 6, 7, 8, 9, 10 |
| 2 | 16 | Yes | Yes | Yes | Yes | Yes | No | Yes | No | + | No | No | 5, 6, 7, 8 |
| 38 | 18 | Yes | No | No | No | No | No | No | Yes | + | ++ | No | No |
| 54 | 19 | Yes | NS | NS | No | NS | No | No | Yes | ++ | +++ | No | 5, 6, 11, 12 |
| 29 | 20 | Yes | Yes | Yes | Yes | Yes | Yes | No | Yes | ++ | + | No | 4, 5, 6 |
| 58 | 26 | No | No | No | No | Yes | Yes | Yes | No | ++ | +++ | No | No |
| 71 | 27 | Yes | No | No | Yes | Yes | Yes | No | No | + | + | No | 9, 10 |
| 22 | 30 | No | No | No | Yes | Yes | Yes | No | No | +++ | No | No | R: 2, 3, 4, 5, 6, 7, 12. L: 2, 3, 4 |
| 35 | 30 | Yes | No | No | No | Yes | No | No | No | No | +++ | Lumbar spine | No |
| 53 | 30 | Yes | Yes | Yes | Yes | Yes | Yes | No | No | + | +++ | No | 2, 3, 7, 8, 12 |
| 37 | 40 | Yes | Yes | Yes | Yes | Yes | Yes | No | No | No | +++ | No | No |
| 36 | 48 | No | No | No | Yes | Yes | Yes | No | Yes | ++ | +++ | No | 2, 6 |
| 47 | 50 | Yes | No | No | Yes | Yes | Yes | No | No | + | +++ | No | 9 |
| 64 | 55 | Yes (small) | NS | NS | No | No | No | No | No | + | +++ | No | No |
| 5 | Adult | Yes | No | No | No | Yes | Yes | No | Yes | ++ | ++ | No | 2, 3, 4, 5, 6 |
| 10 | „ | Yes | No | No | Yes | No | No | No | No | + | +++ | No | No |
| 21 | „ | Yes | NS | NS | Yes | Yes | NS | No | No | + | +++ | Lung | 2, 6, 12 |
| 32 | „ | Yes | NS | NS | No | Yes | No | No | No | No | +++ | No | No |
| 65 | „ | Yes | No | No | Yes | Yes | Yes | No | Yes | + | +++ | No | 9, 10, 11, 12 |
| | | | | | | *Differentiated carcinoma* | | | | | | | |
| 78 | 15 | Yes | NS | NS | Yes | Yes | No | No | Yes | + | +++ | No | No |
| 73 | 37 | Yes | Yes | Yes | Yes | Yes | No | No | No | + | +++ | No | No |
| 72 | 40 | Yes | Yes | Yes | Yes | Yes | No | No | Yes | + | +++ | Liver, rib | No |
| 42 | 48 | Yes | No | No | No | No | No | No | No | + | + | No | No |
| 20 | 50 | Yes | No | No | Yes | No | No | No | No | + | +++ | No | 6 |
| 31 | 70 | Yes | NS | NS | No | Yes | No | No | No | + | +++ | T10 | No. Brachial plexus |
| | | | | | | *Unclassified carcinoma* | | | | | | | |
| 3 | 30 | Yes | NS | NS | Yes | Yes | Yes | No | No | + | +++ | No | 5 |
| 50 | 30 | Yes | Yes | No | Yes | Yes | No | No | Yes | ++ | +++ | Subcutaneous | 3, 4, 6 |
| 60 | 50 | No | No | No | No | No | No | No | No | + | No | No | No |
| 30 | Adult | Yes (small) | Yes | Yes | Yes | Yes | No | No | Yes | + | +++ | No | No |
| | | | | | | *Adenocarcinoma* | | | | | | | |
| 55 | 22 | Yes | Yes | Yes | Yes | Yes | Yes | No | Yes | ++ | +++ | No | 5, 6, 11, 12 |
| 70 | 49 | Yes | NS | NS | No | No | No | No | Yes | No | +++ | No | No |
| 46 | 40 | Yes | No | No | Yes | No | No | No | No | + | +++ | No | No |
| 40 | 75 | No | NS | NS | No | No | Yes | No | Yes | + | No | No | 5, 6, 7 |
| | | | | | | *Lymphosarcoma* | | | | | | | |
| 80 | 35 | No | No | No | No | No | No | No | No | + | +++ | No | No |
| 56 | 55 | No | No | No | No | No | No | No | No | + | +++ | No | No |
| 39 | 70 | Yes | No | No | Yes | Yes | Yes | No | No | + | +++ | No | 12. Brachial plexus |

is common. These tumours are slow growing and benign (McKenzie and Sosman, 1924).

*Mucous gland tumour*

There is a rounded, bulky, soft tissue mass with bone erosion of the palate, antrum, greater wing of the sphenoid and the floor of the middle fossa. Minute calcification is present but is rarely visible on the radiographs in these slow growing tumours which tend to recur after treatment (Pattinson, 1961).

*Nasopharyngeal fibroma*

A soft tissue mass is associated with destruction of the basisphenoid, the floor of the middle fossa and the petrous apex. There is characteristic deviation and destruction of the nasal septum with expansion of the nasal cavity by the growing tumour which appears as a soft tissue mass.

*Glomus jugulare tumour of the temporal bone*

Mastoid and petrous sclerosis is associated with destruction of the petrous area, mastoid and squamous temporal with enlargement of the jugular foramen and involvement of the carotid canal. There is erosion of the articular process of the occipital bone with enlargement of the vertebral canal of the first cervical vertebra and destruction of the temperomandibular joint (Kemp Harper, 1957).

### SUMMARY

The radiological appearances of cancer of the nasopharynx of Africans in Kenya is described. These appearances form a syndrome of retropharyngeal soft tissue swelling, cervical gland masses and erosion of the base of the skull. Age is not shown to vary the radiological picture and except for the sarcoma group no relationship is found between the histological appearance and the radiological findings.

I am grateful to Miss Gibson and Miss Khimasia of the Department of Radiology, King George VI Hospital, Nairobi, for their assistance in developing the radiographic techniques used for these patients.

### REFERENCES

CLIFFORD, P.—(1961*a*) *J. Laryng.*, **75**, 707.—(1961*b*) *E. Afr. med. J.*, **38**, 491.
*Idem* AND BEECHER, J. F.—(1964) *Brit. J. Cancer*, **18**, 25.
*Idem*, OETTGEN, H. G., BEECHER, J. L., BROWN, F. P., HARRIES, J. R. AND LAWES, W. E.—(1963) *Brit. med. J.*, i, 1256.
GODTFREDSON, E.—(1944) *Acta path. microbiol. scand.*, Suppl. 50, p. 240.
HO, H. C.—(1961) ‘ Tropical Radiology,’ London (Heinemann).
KEMP HARPER, R. A.—(1957) *J. Fac. Radiol., Lond.*, **8**, 325.
LEDERMAN, M.—(1961) ‘ Cancer of the Nasopharynx,’ Springfield, U.S.A. (Thomas) p. 52.
LIANG PO-CH’IANG, CHEN GIAN-CHING, TSUH JA-TSEN, HWU YUANG-FAN, TSU SHAU-MAN, TSUNG YUNG-SUN.—(1962) ‘ Selected Papers on Cancer Research,’ Shanghai (Shanghai Scientific and Technical Publishers) p. 106.
LINSELL, C. A.—(1964) *Brit. J. Cancer*, **18**, 49.
MCKENZIE, K. G. AND SOSMAN, M. C.—(1924) *Amer. J. Roentgenol.*, **11**, 171.
PATTINSON, J. N.—(1961) *Clin. Radiol.*, **12**, 251.
SHU-YEH.—(1962) *Cancer*, **15**, 895.